# Acknowledgment to Reviewers of *Current Oncology* in 2021

**DOI:** 10.3390/curroncol29020059

**Published:** 2022-01-29

**Authors:** Current Oncology Editorial Office

**Affiliations:** MDPI AG, St. Alban-Anlage 66, 4052 Basel, Switzerland

Rigorous peer-reviews are the basis of high-quality academic publishing. Thanks to the great efforts of our reviewers, *Current Oncology* was able to maintain its standards for the high quality of its published papers. Thanks to the contribution of our reviewers, in 2021, the median time to first decision was 21 days and the median time to publication was 47 days. The editors would like to extend their gratitude and recognition to the following reviewers for their precious time and dedication, regardless of whether the papers they reviewed were finally published:
Aaron MansfieldKay-Dietrich WagnerAbed AgbaryaKazuhito FunaiAbha GuptaKazuhito SuzukiAbhishek ShankarKazumasa MatsumotoAdile OrhanKazuya ShinmuraAditya GanjuKea L. TurnerAdolfo RomeroKeiichiro UeharaAdrian D. SchubertKeith HolmesAgata FilipKelli StajduharAgata PastorczakKenichi SudaAgnieszka Jagiełło-GruszfeldKensuke MitsunariAhmed A. HusseinKerollos Nashat WanisAhmet Emre EskazanKerri BeckmannAiham QdaisatKevin B. KnopfAkihito OkazakiKevin D. BoydAkshara RaghavendraKevin ZarrabiAlan AstrowKhaled ZazaAlan BatesKian KaniAldo BoveKimberly E. StephensAlessandro ComandoneKinan Drak AlsibaiAlessandro De VitaKirtikar ShuklaAlessandro RizzoKit WongAlessandro RussoKlazien Matter-WalstraAlessandro ValdegamberiKoji AndoAlessia PellerinoKonstantinos PorpodisAlexander E. KabakovKosuke TakemuraAlexander P. ColeKouichiro KawanoAlexander V. BagaevKristen HaaseAlexander V. LouieKrystyna PawlakAlexander WinterKrzystof TomasiewiczAlexandra CaziucKuo-Hsin ChenAli BoolaniKurtis EisermannAli Salehzadeh-YazdiKyle VanKoeveringAlice IndiniKyuwan LeeAlicja FormaLaith R SultanAlireza GhoreifiLászló Csaba MangelAmanda PiperLaurel O. SillerudAmbica ParmarLaurentiu PopAmirrtha SrikanthanLaurie ZawertailoAmit Satish BhattuLayth Mula-HussainAmy S. DeGroffLeandro SastreAna Isabel Fraguas-SánchezLei ShiAna LohmannLeif Hendrik DrögeAna Luísa De Sousa-CoelhoLeila JahangiriAna Mendes LealLeonard Christopher SchmeelAna Rita CarlosLesley AndersonAnastasios KarayiannakisLetizia DeantonioAnat BiegonLindsey DayerAnca SimionescuLisa BashoreAnders Erik Astrup DahmLisa CunninghamAnders Husted MadsenLisa M. PickeringAndrada SeiceanLisardo BoscaAndre MoreiraLiselle BovellAndré VisLiudmila V. SpirinaAndrea BellieniLizz Van Der HeijdenAndrea C. LoLokeshwar VinataAndrea Giuseppe MaugeriLoredana MarcuAndrea PatriarcaLorenzo AndreaniAndrea SambriLorenzo AntonuzzoAndrea VodermaierLorenzo BelluominiAndreas Wolfgang ReskeLorenzo BianchiAndrew HardingLorenzo SpaggiariAndrew RedfernLori Anne WoodAndrey ElchaninovLouisa BolmAngel ArnaoutLouise HillerAngel Romero ColladoLuca CalabreseAngela MastronuzziLuca FilippiAngeline LetendreLuca GiacomelliAnil GumberLuca Giovanni LocatelloAnkit ManglaLuca Lo NigroAnna BartosiewiczLuca MoscettiAnna GagliardiLuca NespoliAnna Maria FrustaciLuca Oscar Redaelli De ZinisAnna Myriam PerroneLucia A. PossamaiAnna Nasierowska-GuttmejerLucian Constantin MocanAnna PecorelliLucie Lafay-CousinAnna RotiliLucie M. TurcotteAnna StarzyńskaLucio MangoAnna SynakiewiczLui NgAnne Marie RuppertLuigi BennardoAnne Sophie BaudryLuigi De MariaAnne W. BeavenLuigi RigacciAnnemarie Leliveld-KorsLuis Alberto Pérez RomasantaAnne-Sophie WoznyLuís Pacheco-FigueiredoAnnette HayLuis Pérez BelmonteAnteo Di NapoliLuis RodrigoAnthea C. PetersLuisa Matos Do CantoAnthony FieldsLukas BunseAnthoula LazarisLukasz WicherekAntoine Bouchard-FortierLynn F. LavalleeAntonella CacchioneLyudmila Bel'skayaAntónio AraújoMagnus HultinAntonio BiondiMahmoud KhalifaAntonio De LeoMakoto EndoAntonio Di MeglioMalcolm H. SquiresAntonio GalvanoMalek B. HannoufAntonio GiorgioMallorie B. HeneghanAntonio Giovanni SolimandoManfred P. LutzAntonio Mario ScanuManoj KandpalAntonio MatroneManuel MonrealAntonio PassaroManuela RabaglioAntonio PercesepeMarcin NicosAntonio Sarria-SantameraMarco AgusAntonio SommarivaMarco FalascaAntonio ToescaMarco IafollaAntonio VallanoMarco Lorenzo BonùArash AsherMarcus U. HentrichArash NavranMarcy WingetArcangelo SebastianelliMarek BugajskiAri MeguerditchianMargaret FitchArnaud PagesMargit L.H. RiisÁrpád SzállásiMaría A. Pérez-HerreroArtur SłomkaMaria Adele MarinoAshley Barry GrossmanMaria C. KatapodiAtsushi MakimotoMaria Cristina De MartinoAttila ZalatnaiMaria Gabriela O. FernandesAtul BatraMaria GrauAudrey RousseauMaria IkonomopoulouAurora MaurizioMaria KapritsouAzadeh ElmiMaria LavdanitiBahil GhanimMaria Lucia Hirata KatayamaBalaji KrishnamacharyMaria PanagopoulouBarbara-Anne MaierMaria VolkovaBarliz WaissengrinMariangela ManciniBartłomiej TomasikMarieke StraverBartosz MałkiewiczMarika QuadriBeata Kasztelan-SzczerbińskaMarina AgozzinoBeata Szulc-MusiołMarina Letica CrepuljaBeatrice Gabriela IoanMarinela BostanBefikadu WubishetMario De FeliceBehrus PuladiMário EspadaBelinda GoodwinMario PirisiBenedetta GuaniMarion RappBenjamin HengMarius RademakerBenjamin L. LampsonMariusz BidzińskiBenjamin L. MaughanMariusz DąbrowskiBenjamin LardinoisMark P. LythgoeBenjamin RybickiMark VincentBeomseok KoMark WunderlichBerardino De BariMarko JakopovicBernhard MeyerMarkus HoffmannBertrand PorroMarkus KallerBertrand RoutyMarshall PitzBeth MallardMartin BarrBeverly LongMartin C. TammemägiBignardi MarioMartin RutegårdBijal D. AminMartin VlkBishal GyawaliMartina LambertiniBleddyn JonesMartina SilvestriBo GaoMartine CrosetBonnie LeungMartine J JagerBoshra ArnoutMartine T.E. PutsBrandon A. HowardMary Munaza TajBrett Malcolm ColdironMaryam DosaniBrian Andrew Van TineMasaharu HataBrian KellyMasahito NakanoBrian SamuelsMasatsugu IwamuraBrian ShawMasaya IwamuroBruno FiondaMasayoshi FujisawaBryan WinnMassimiliano CretaByeongsang OhMassimo GentileCagatay GünesMassimo LibraCaitlin C. MurphyMassimo MascoloCaitlin TakahashiMassimo NabissiCaitriona CahirMassimo OffidaniCandice JohnstoneMateusz Jacek SpałekCarl J. BrownMateusz SpałekCarla PisaniMatteo CrippaCarlo Augusto MallioMatteo De PastenaCarlos Vaz De CarvalhoMatteo GhilliCarmen Griñán-LisónMatteo SuterCaroline MarianoMatthew DixonCaroline VerbekeMatthew MeiCarolyn OwenMatthew PainschabCaterina SoldàMatthew S. PainschabCatherine LabbéMatthew Stuart McKinneyCecilia AriciMatthias MillesiCédric CoulouarnMatthias SteinertCeleste CagnazzoMattia Falchetto OstiCelia Marti-GarciaMattia RiefoloChaitanya ValivetiMattias SandströmChandra Sekhar BathulaMauro AlaibacChaoHua ChiuMd MohiuddinChara StavrakaMeiling YapCharles L. ShapiroMelania CarlisiChellappagounder ThangavelMelanie MorrisChelsea MayohMelissa L. HillCheng Hong TsaiMelissa SoutheyChenghui LiMeredith GiulianiChenyu SunMetis HasipekCherie P. ErkmenMichael B. MannCheryl HoMichael C. BurgerCheryl PetersMichael ChrobokChi Lam Au YeungMichael EngelbertChiao-En WuMichael GrunertChiara Maria MazzantiMichael HalpernChia-Sui WengMichael I. KoukourakisChiau Ming LongMichael J. GraylingChienan LiuMichael K. FarrisChien-Feng LiMichael Michael SamarkosChihiro InoueMichael OsbornChih-Jen YangMichael V. JaglalChihyang WangMichael WelshChing-Hu ChungMichail PlotkinChing-Sheng HsuMichel KalamaridesChloe E. AtreyaMichele GhidiniChristian FurthMichelle J. HendersonChristin B. DeStefanoMichelle MollicaChristina M. Dieli-ConwrightMichelle NadlerChristina Maresch BernardesMihnea-Alexandru GamanChristina WuMilad MoloudizargariChristine J. FarrMilada ZemanovaChristine MarosiMin H. HuangChristoph ThomssenMineko TeraoChristopher BoothMireille A. NorrisChristopher CaoMohammad MohammadianpanahChristopher J. LongoMohammad SaadChristopher LoMomcilo JankovicChristopher MelaniMonika Klinkhammer-SchalkeChristopher Paul VennerMonika SobočanChristos ChouaidMonish Ram MakenaChristos IavazzoMoran AmitChristos K. KontosMotohiro HiraoChristos MikropoulosMuhammed KibriyaChristos TsalikidisMurat GokdenChrysanthi KarapantzouMustafa Zelal MuallemChunchia ChengNadia NajafiChung-Chien HuangNadine S. AguileraChung-Chun WuNageswari YarravarapuChun-Nan YehNajme JafariClaire F. Temple-OberleNakhle S. SabaClaire M. B. HollowayNam NguyenCláudia Alhinho MouratoNancy A. NixonClaudio FeoNao FujimoriClaudio LuchiniNaomi DolgoyClemens F. M. PrinsenNarcyz GhineaCliff J. LukeNaseer AhmedColleen M GallagherNatasha KekreConcetta QuintarelliNatasha T. HillCorina Kim-FuchsNatasja RaijmakersCorinne PrévostelNatsumi FujiwaraCosimo CumboNenavath Gopal NaikCostantino ErraniNéstor MartínezCraig ErkerNeville HackerCristina ChircovNguyen Minh DucCristoforo SimonettoNibedita PatelDagmara KłopotowskaNiccolò PetruccianiDagmara Szmajda-KrygierNicholas O. MarkhamDaniel A. MulrooneyNick PavlakisDaniel B. StovallNicol StrakováDaniel BreadnerNicola Luigi BragazziDaniel López-LópezNicola MaggialettiDaniel MeyersNicola MontemurroDaniel Moreira-GonçalvesNicolae GicaDaniel RaysonNicolas MarcouxDaniel WieseNicole MifsudDaniela MarazzitiNicole RankinDaniele DondossolaNicolòmaria BuffiDaniele La ForgiaNigel J. AndersonDaniele VergaraNigel S. B. RawsonDanilo FioreNikolaos MachairasDannel YeoNikolay A. PyataevDario Di StasioNikolay MehterovDario TrapaniNina Milutin GašperovDarren Wan-Teck LimNitin RoperDavid AnnNoah SamuelsDavid D. EisenstatNong XuDavid FornerNormand BlaisDavid H. AggenNuno BernardesDavid J. StrausNuno Jorge LamasDavid MathieuNurbubu MoldogazievaDavid NussbaumOlaf SchofferDavid S. BaskinOlga BureninaDeborah J. BowenOlga OikonomidouDebottam SinhaOlivier PeyronyDebra J. BoardleyOlivier Q. GrootDemin CaiOmar AlhalabiDenis SoulieresOttavia Eleonora FerraroDerek A WainwrightPablo MandóDhananjaya KulkarniPalanisamy NallasamyDianne SheppardPamela HebbardDiego F. CalvisiPamela PetersDijana DjureinovicPanagiotis G. HalvatsiotisDimple ChakravartyPanagiotis J. VlachostergiosDina Di GiacomoPanagiotis TsagozisDina El DemellawyPaola TombesiDinesh PradhanPaolo PrandoniDivya RamchandaniPaolo SpinnatoDomenico AlbanoPaolo TarantinoDomenico FerraioliParis-Ann IngledewDomenico FuscoParita RatnaniDomenico GalettaParneet CheemaDominik RadzkiPasquale CianciDominika GuzekPasquale PisapiaDonatella BrancorsiniPasquale ZizzaDoreen EzeifePasquale EspositoDouglas A. ArenbergPatrice ForgetDouglas E. NeyPatrick M. BolandDusan HrckulakPatrick NasarreDusana MajeraPatrizia FerroniEdmond RizcallahPaul CarrierEduardo FrancoPaul Van HoutteEdyta Maria UrbanskaPaul Wheatley-PriceEdyta Marta BorkowskaPaula BeneveneEgidio CelentanoPavlos MsaouelEiichi IshikawaPavol GenzorEiji KiyoharaPaxton V. DicksonElena RizaPedro Miguel RodriguesElias KehagiasPernille HolmagerElisa GenuardiPetar OzretićElisabeth AndritschPetca Razvan-CosminElisabeth CoynePeter M. AndersonElisabeth Nieveen Van DijkumPetros SountoulidesElizabeth KertowidjojoPetter GjersvikElizabeth S. DylkePhyllis ButowEllen CarlPia Lopez-JornetEllen GreenblattPierfrancesco FrancoElżbieta CiporaPierre Yves R. MarcyEmad Al-DujailiPietro FerraraEmese GellénPietro Luigi PolianiEmese MezõsiPietro QuaglinoEmilio Francesco GiuntaPietro SpennatoEmmanouil SpanoudakisPilar López-NievaEnrica Teresa TandaPiotr DzięgielEnrico CassanoPiotr KornetaEnrico FioriPiotr Mirosław MaŁkowskiEnrico MarinelliPovilas IgnataviciusEnrique Gomez GomezPrafull GhatageErdem GökerPranshu SahgalErez Bar-HaimPritam SadhukhanÉric ChastrePrzemyslaw PlonkaEric JeandidierQiangzhe ZhangEric SmithQuincy ChuErnestas JanulionisRachel GoodwinErtuğrul ŞahinRadka VáclavíkováEsther DajczmanRafael RosellEun-Joo ParkRafał PęksaEvangelista LauraRafał WatrowskiEvgenia A. HalkiaRaffaele FrazziEwa Nowakowska-ZajdelRaffaella PalumboEzekiel WeisRahul LakhotiaFabian Martin TroschelRahul MannanFabien ForestRajeev PandeyFabrizio SignoreRaksha BhatFahim AhmadRalph MeyerFAISAL AZIZRavi P. SahuFalk RoederRavi RamjeesinghFang WeiRavindran Caspa GokulanFausto MaffiniRebecca HarrisonFay StrohscheinRegis Resende PaulinelliFederico FerrariRicardo FernandesFederico NalessoRicardo LeãoFelice CrocettoRiccardo DraghiFelipe NörRiccardo MastroianniFengchao LangRichard OwenFeng-Ming HsuRikio SuzukiFerdinando AgrestaRintaro HashimotoFerdinando Carlo Maria CananziRio Ryohichi SugimuraFerdinando Carlo SassoRita FranciscoFernando De MoraRobert A. KurtFernando HernanzRobert D. KilgourFiona Crawford-WilliamsRobert G. BestFlorian GesslerRobert MandicFlorian LangerRobert Steven EsworthyFlorin GraurRoberta ModicaFoteini MalliRoberto CasconeFoteinos-Ioannis DimitrakopoulosRobin PierceFraia MelchiondaRobyn MacfarlaneFranca FagioliRocío BenitoFrances Mary JohnsonRocio Eiros BachillerFrancesca DuraturoRocío TalaverónFrancesco CucciaRoger HenrikssonFrancesco D'AlòRohan SharmaFrancesco Di MolaRolf HolleFrancesco LeoRolf SøkildeFrancesco RestelliRonald A.M. DamhuisFrancesco SchettiniRonald BurkesFrancesco TovoliRonald D. BarrFrancisco J. EnguitaRosa María López-Pintor MuñozFranck BillmannRosario CaltabianoFrancois DionneRossi MicheleFrank ClaessensRuchi ShuklaFrank EssmannRuixue HouFrank FrizelleRupert BartschFrank KnoefelRuth Birner-GruenbergerFranz Joachim HilkeRuth LewisFranz ZehentmayrS. G. AfanasyevFrederic LezotSaadi KhochbinFuchsia HowardSaeed SadeghiFulvio BorellaSafiya KarimFumiaki IsohashiSahar SalehiFumitaka KogaSaima ImranGábor RubovszkySakti ChakrabartiGabriela AniteiSally E. ThorneGabriela IlieSally LauGabriele TudertiSalvatore Massimiliano CardaliGaetano AurilioSalvatore SiracusanoGaetano RiemmaSameer GopalaniGąsowska-Bajger BeataSami Abd ElwahabGautam MehtaSamrita DograGautam N. ShenoySamuel Peña-LlopisGautam SethiSamuel SamnickGeeta SharmaSan Ming WANGGeneviève C. DigbySanchia ArandaGennaro CormioSandeep K. SinghalGeoffrey CrispSang-Han LeeGeorden JonesSara PączekGeorg StüssiSara PizzamiglioGeorge DranitsarisSarah MougalianGeorge ShenoudaSarah Nicole HamiltonGeorgia HalkettSarel HalachmiGerald ClamonSarfaraz HusseinGerardo FerraraSarmad H. JassimGergely VargaSasha LupichukGhanim KhatibSatoru MiuraGiacomo AndreaniSaúl Oswaldo Lugo ReyesGiacomo BregniSazan RasulGian Paolo CavigliaSeamus O’ReillyGiancarlo FacchiniSebastian KrugGianluca SpitaleriSebastian SzmitGiedre SmailyteSelena MimmiGiorgio GinesuSelene Meza-PerezGiovanni BarillariSeongho KimGiovanni CrisafulliSerafin MoralesGiovanni GrignaniSerena CeddiaGiovanni TazzioliSergei BoichukGiulia FerrarazzoSergio BertoglioGiulia M. StellaSergio CortelazzoGiuliana MuzioShaila J. MerchantGiulio RossiShaoan WangGiuseppe AngelicoShao-Hsuan KaoGiuseppe FanettiSharon WatanabeGiuseppe GiudiceSharyl NassGiuseppe MarulliShashank SrivastavaGiuseppe Roberto GiammalvaShazid Md. SharkerGiuseppe SammarcoShebli AtrashGiuseppe SchepisiShekoufeh AlmasiGiuseppe StragliottoShelize KhakooGlenn BaumanSheng-Mou HsiaoGopichand PendurtiShigehiro KarashimaGreg M. TiaoShigehiro KokubuGregor NorcicShigeo OhbaGregor SeršaShihlung ChenGregor StavrouShin NishioGregory Bruce MannShinichi KinamiGrégory VerhoestShinji KawabataGuido CostaShinji TsukamotoGuido GiordanoShintaro NaritaGulam RatherShinya MatsuzakiGunjan ShahShiv VermaGunnar KristensenShiying LiGünter EmonsShun-Fa YangGuy FroyenShu-Pin HuangGyozo SzolnokyShu-Yan LiH. Denis AlexanderSiddharth ShethHabeeb MajeedSimona BorstnarHaitham JahramiSimona TavernaHåkon ReikvamSimone MorraHammad GanatraSimone PolvaniHank HoSimone SerafiniHanna KnausSonali P. BarweHannes LueckingSonam MittalHannnah-Rose MitchellSophie LebelHans ChristiansenSophie LelorainHans G. DrexlerSoumyajit D. RoyHans JunkermannStacey K. MardekianHao ZuoStanley L. LiauwHaris A. CharalambousStefan NilssonHarun FajkovicStefan ScheibHavva Yeşil ÇınkırStefan SiemerHelena PópuloStefania CrucittaHenk K. Van HalterenStefano DastoliHenry S. ParkStefano LuzzagoHerwig CerwenkaSteffen WagnerHester Van De BovenkampStephan BuseHideaki KawabataStephanie SnowHideyuki FukuiStephen FalkHimangshu SonowalStephen YipHiroko NishidaStergios BoussiosHiroko YamashitaSteven MutsaersHiromichi ItoSteven Paul NisticòHiroshi FukushimaSubrata DebHiroshi MiyamotoSukeerat RubaHirotaka MatsuiSuk-hwan LeeHirotaka NishiSuman DuttaHiroyuki SasaiSumitra MohanHisham BahmadSung Hee LimHoman MohammadiSunita GhoshHong FangSusanne M. ArnoldHong Sheng ChengSuzanna ZickHooshang LahootiSvetlana Bajalica-LagercrantzHossein NikzadSylvie LambertHsiang-Cheng ChiSynnøve YndestadHsien-Bin HuangSzu-Tah ChenHugo Arasanz EstebanTadeusz PawelczykHugues JacqminTahir ShahHui-Hua HsiaoTai HatoHung-Wei WangTakafumi SatoHung-Yi ChuangTakahiro InoueHyun Jo YounTakahiro KogawaHyung-Soo HanTakamichi ItoIdoia Martín-GuerreroTakashi NomizoIe-Bin LianTakeaki IshizawaIgor AurerTakehiro IwataIgor ShendrikTakehiro OtoshiIngrid LilienthalTakemi OtsukiIoannis VoutsadakisTakeo NakadaIrina Gepfner-TumaTakeshi SasakiIris ForteTaketo YamadaIsaac RuizTakuji HayashiIstván KissTakuya KoieIulia AndrasTami BornemanIvana PeranTao FuIveta SelingerovaTara BaetzIvica RatosaTara HorrillIwona Głowacka-MrotekTarek BismarIwona J. GisterekTariq MalikIwona MalickaTengchuan JinIzabela ChmielewskaTeresa PerraJaafar BennounaTerry C.F. YipJacek J. SznurkowskiTessa BrabanderJacek SzeligaTetsuhiro YoshinamiJacob FreedmanTheodoros VassilakopoulosJacopo Junio Valerio BrancaThierry AlcindorJacopo NoriThierry Oscar EdohJakob K. AnningaThomas E. MürdterJames KuoThomas F. MonaghanJames MainprizeThomas J. SmithJames MichaelThomas PabstJan FrankoThomas SalopekJan JežekThomas Wesley TempletonJanice A. BlalockTimm DeneckeJann ArendsTimothy AsmisJanos G. PitterTimothy HannaJared J. BarrottTina ŽagarJaromir AstlTine NordgreenJarosław ĆwikłaTish KnobfJarosław KobielaTom J. H. ArendsJar-Yi HoTomáš BüchlerJasmina LukinacTomas JelinekJasminka OmerovicTomas KoltaiJason X. ChengTomasz MarjanskiJason Yongsheng ChanTomoki NakamuraJavier Menéndez-MenéndezToralf ReimerJavier Molina-CerrilloTorill SauerJavier VaqueroToru HiragaJayant S. VaidyaTsuyoshi FukushimaJean L. KoffTsuyoshi KobayashiJeanette ReeceTyler ChesneyJee Hyun KongUgo FalagarioJee Soo ParkUlrike BacherJeffrey BuckleyUmberto MalapelleJeffrey CaoVaishnavi MuralikrishnanJeffrey GimbleVakul MohantyJeffrey R. AndolinaValentina CossigaJennifer J. GariochValentina I. PetkovJennifer PhilipValentina LancellottaJennifer SchneiderValentina RossiJennifer ShengValerio Di PaolaJenny BrandsValerio GallottaJenny QueVangala ChandanJens Ahm SørensenVania Balderrama BrondaniJens HahneVeronica AranJens J. RassweilerVeronica JonesJens JakobVeronika HuntosovaJeonghyun KangVictor YazbeckJer-Hao ChangViet LeJerzy BeltowskiVincent Thomas-de-MontprévilleJerzy ChudekVincent Van Der NoortJezabel Rodriguez-BlancoVinodh KakkasseryJie LeeVirginia ValentiniJiefu JinVita GolubovskayaJill Marie KolesarVítor CavadasJillian R. GüntherVittorio GebbiaJin Hyoung KimVlad Laurentiu DavidJin ZhangVolker ArndtJing-Quan WangVyshak VenurJin-Ming WuWai-Lung NgJin-Yuan ShihWanlin JiangJiong LiWee Loon OngJoanna CyrtaWei-hsiung YangJoão CastelhanoWei-Ting ChaoJoaquim CarrerasWeiwei ZhangJoelle T. FathiWen Tsan ChangJohanneke PortieljeWendy R. ParulekarJohn GriniatsosWenjun WuJohn HiltonWillemijn SME TheelenJohn M. PerryWilma HopmanJohn Thomas LucasWinston LiauwJonathan I. IzawaWi-Young SoJoo Ha HwangWon-sik HamJoost Van GriethuysenWoo Chul NohJordi MuntaneWoo Kyung JeongJordi RemónWouter W. De HerderJörg KleeffXiuhua WengJörg LindenmannYao-tseng ChenJorge CervantesYasmin Lassen-RamshadJorge DazaYasuhiro OkumuraJosé Javier Zamorano-LeonYasunobu YamashitaJosé-Ángel Hernández-RivasYen-Hao ChenJoséJ Juan IllarramendiYen-Kung ChenJoseph KwongYeonok SuhJoseph M. ConnorsYi-Jang LeeJoseph OjahYing-Cheng ChiangJoseph VadakaraYingzi ZhangJoshua J. WaterfallYoji KishiJosko BozicYoonsun ChungJuan Carlos De VicenteYoshifumi IwagamiJuan GaristoYoshikane YamauchiJuan P. RodrigoYoung ChoiJudy NeedhamYoung-Joon JunJue JiangYoung-Youn ChoJulia BurnierYousuke NakaiJulian KenyonYu Jin LimJulian PohlanYu-Chan ChangJulie LemieuxYu-Ching LinJumin ParkYuichi TamboJun KiuchYuko HaradaJun TeishimaYun-Chung CheungJung-Ju HuangYun-Shiuan ChuangKa Yu TseYuta ShibamotoKaeko KameiYuto YasudaKaining ZhiYvonne EngelsKalyan SaginalaZachary VeitchKamisha EscotoZahra MojtahediKanhua YinZara SteinmeyerKaren A. GelmonZbigniew JabłonowskiKariem SharafZélia VelezKarin WelenZenia GhorabKarine MichaudZgura AncaKarl RiabowolZhen QiuKarolina KowalskaZhenghong LeeKatarzyna J. JerzakZhijun ZhouKatarzyna KuśnierzZhiyun XueKatja MohorcicZilvinas DambrauskasKatja SteigerZiya L. GokaslanKatsuhiro Ito



